# Post-measurement compressed calibration for ICP-MS-based metal quantification in mine residues bioleaching

**DOI:** 10.1038/s41598-022-19620-8

**Published:** 2022-09-26

**Authors:** Beatriz Rito, Diogo Almeida, Carina Coimbra, Diogo Vicente, Romeu Francisco, Rita Branco, Harald Weigand, Paula Vasconcelos Morais

**Affiliations:** 1 Centre for Mechanical Engineering, Materials and Processes, Department of Life Sciences, University of Coimbra, 3000-456 Coimbra, Portugal; 2 Competence Centre for Sustainable Engineering and Environmental Systems, THM University of Applied Sciences, Wiesenstr. 14, 35390 Giessen, Germany; 3 Present Address: School of Chemistry, University of St. Andrews, Fife, Scotland, UK

**Keywords:** Environmental sciences, Environmental biotechnology, Scientific data

## Abstract

Bioleaching is an actual economical alternative to treat residues, which allows, depending on the chosen strategy, two possible outcomes: (1) a leachate enriched with target metals, or (2) a residue enriched in target metals through the leaching of interfering components (IC). This work aimed to study the metals released by bioprocessing the Panasqueira mine tailings, as a strategy to increase critical metals' relative concentration in residues. Biostimulation of the local microbiota was compared to a bioaugmentation approach using the autochthonous *Diaphorobacter polyhydroxybutyrativorans* strain B2A2W2. Inductively Coupled Plasma Mass Spectrometry (ICP-MS) was selected to study the metals released in the leachate through multi-element external standards. A new data treatment method was developed to use a preliminary sweep of intensities to quantify the non-initial target metals concentration in the leachate, based on preliminary ICP-MS intensity measurements. The results demonstrated that biostimulation was an efficient bioleaching strategy for the IC silicon, aluminium, magnesium, selenium, manganese, zinc, iron, and copper, by decreasing concentration, resulting in a relative increase in the gallium and yttrium (10x) levels in the treated residue. The strategy followed to quantify a large number of elements with ICP-MS using a reduced number of data points for calibration proved valid and speeded up the analytical process.

## Introduction

Continuous population growth, elevated levels of economic activity and technological innovation are all driving a rapid increase in resource consumption, making sustainable resources use an imperative issue for humanity^1,2^. Therefore, in the last years, new ideas on materials use and circularization have been raised^3^. Traditional mining activity produced residues that were deposited in mine tailings basins, which contain metals as byproducts of the extraction processes^4^. In the past, those processes were not as efficient as today and, in consequence, the mining tailings can be considered low-grade ore^5,6^. Thus, in a circular economy concept, innovative methods and processes for the efficient extraction of metals are required^7^. With this in mind, biological approaches emerged since they can be more cost-effective and environmentally friendly when compared to chemical treatment of mining residues. New bio-approaches such as bioleaching are seen as promising technology for the future and may contribute to a zero-waste society^8,9^.

Bioleaching, as a hydrometallurgical technique, takes advantage of microorganisms to promote the extraction of metals and metalloids from solid material^8,10^. Despite the tailings’ metal concentrations and consequent environmental toxicity, several works have described the presence of metabolically active autochthonous microbial communities^11,12^. This diverse microbial community in mine tailings is considered to play an important ecological role and promotes several metal interactions^13^. Metal recovery from sulfide minerals is usually based on the activity of chemolithotrophic bacteria, mainly *Acidothiobacillus ferrooxidans* and *A. thiooxidans*, which convert insoluble metal sulfides into soluble metal sulfates. This is the most explored strategy already applied by different companies worldwide. Heterotrophic bacteria as well as fungi have been used lately to treat non-sulfide ores and minerals. In these cases, metal extraction is due to the production of organic acids and chelating and complexing compounds excreted into the environment. Strains from the genera of *Pseudomonas*, *Bacillus* and *Rhizobium* are among the most powerful bioleachers heterotrophic bacteria^14^.

Compared to batch shaking tests, dynamic column experiments more closely mimic the bioleaching processes that are effective under real conditions (heap-scale)^15–17^. In the last years, several works have been published on the evaluation and analysis of residues and their reuse^18^. In this regard, Munoz and collaborators^19^ demonstrated that copper bioleaching in column reactors provided more useful information on the effect of various parameters (particle size, ore type, and substrate) than shake flask studies toward a heap-scale application. Previous works also showed that the efficiency of bioleaching is affected by different parameters of the contaminated soils and tailings, including pH, temperature, medium composition, particle size and bulk density^20,21^. Zhang and collaborators^8^ confirmed that organic acids, pH and siderophores contributed to rare earth elements (REE) leaching, although the relative importance of these parameters was strain-specific. On the other hand, Hao and collaborators^22^ achieved 60% of copper extraction on columns bioleaching, verifying that solution pH as well as ferric iron significantly influenced the microbial community.

As mentioned above, bioleaching experiments usually focus on the quantification of metals that are transferred by bacteria from the solid phase into solution or determine the change in residue composition by X-ray Fluorescence (XRF)^23^. Obtaining pore water samples is relevant since biological metal mobilization occurs at the micro-level between the bacteria and the ore grain^24,25^. Leaching may be considered the result of gradients in constituent concentrations between the pore water solution of the solid and the contacting water collected by percolation^26,27^.

Elements present in the leachate are related to the composition of the residue and can be a fraction, or the totality, of the elements present in the latter^26^. The bioleaching in presence of the tailings’ autochthonous microbial community may also be performed by biostimulation, which aims at the activation of several, not completely known, metabolic pathways and in consequence results in leachates with an a priori unknown composition^28^. Previous works have bioinformatically estimated the metabolic parameters that could be involved in biostimulation^13^. On the other hand, bioaugmentation strategies use characterized autochthonous bioleachers for the leaching of target metals^29^.

In this work, in a first approach, the release of tungsten (W), a critical raw material according to the European Commission^30^ was followed by both biostimulations of the autochthonous microbial community and by bioaugmentation with an autochthonous strain, under different conditions. Inductively Coupled Plasma Mass Spectrometry (ICP-MS) was used to study the metal release in the leachate during the incubation period^31^. This technique was chosen primarily due to its high accuracy, low detection limit and the possibility to use multi-element external standards^32^, which allows for the calibration of a greater number of elements. New insight into the bioleaching potential of the used microorganisms was obtained by following the content of critical target metals in the leachate. A relevant concentration of non-target metals was detected in the leachate. The mixture of these interfering components (ICs) i.e. iron (Fe), silicon (Si), aluminium (Al), zinc (Zn), copper (Cu), manganese (Mn) and magnesium (Mg), was quantified by ICP-MS from the intensity measurements using a new data treatment method and a modified calibration model, both described and tested in the current work. On the other hand, the target metals were quantified by calibration curves through the usual peak-hopping precise determination. The leaching of IC could lead to the enrichment of critical target metals in the residues, attributing relevance to the ICs quantification.

## Materials and methods

### Tailings sampling and sample pre-treatment

Mine tailings samples were collected at 0.5 m depth from Panasqueira Mines’ Basin 1 (B1)^13^ and kept at 4 ºC during transportation and until use to preserve the autochthonous microbial community. Before the experiments, the mine tailings (grain diameter 1—10 µm) were mixed in a 1:1 ratio with quartz sand (grain diameter 100 – 300 µm) to reach a final mass of 3.0 kg. This tailings admixture was performed without drying. The tailings composition and metals’ final concentration in the residues were already described in previous work and are summarized in the supplementary material (Supplementary Table S1 and S2)^13^. The tailings contained mostly Si (27,400 ppm), and relatively high amounts of W (1,470 ppm), Al (105,000 ppm), Zn (4,431 ppm), Cu (1,960 ppm) and Fe (54,946 ppm). The total organic carbon (TOC) in the sediments was lower than 1%. The pH varied between 6 and 7 and presented low to uncertain net acid generation (NAG) potential. The autochthonous microbial community present in the B1 tailings was mainly composed of Proteobacteria (59%) and Firmicutes (34%).

### Bacterial isolate, identification and culture conditions

*Diaphorobacter polyhydroxybutyrativorans* strain B2A2W2, a gram-negative and facultatively aerobic bacterium, was isolated from Panasqueira mine tailings in Basin 2 (B2)^13^ in Reasoner’s 2A (R2A) medium (Oxoid) containing per liter 0.5 g yeast extract, 0.5 g peptone, 0.5 g casamino acid, 0.5 g glucose, 0.5 g starch, 0.3 g dipotassium phosphate (K_2_HPO_4_), 0.05 g magnesium sulfate heptahydrate (MgSO_4_.7H_2_O), and 0.3 g sodium pyruvate). This strain was cryopreserved at the University of Coimbra Bacterial Culture Collection (UCCCB), at -80ºC, in Tryptic Soy Broth with 15% glycerol. The characterization of the strain to be included in the culture collection showed *D. polyhydroxybutyrativorans* B2A2W2 ability to produce siderophores and its genome analysis demonstrated the presence of organic acids extrusion and production pathways. The biomass for bioaugmentation experiments was produced by growing the bacterial strain in R2A broth (R2Ab) medium (Himedia) in a batch system at 25 ºC, overnight, at 125 rpm. The culture was centrifuged to remove the growth medium and recover the pellet cells. The bacterial suspension (100 ml) was then prepared by resuspending the cells to an optical density of 600 nm (OD_600_) of 0.1 in R2Ab. This bacterial suspension was used to inoculate the bioaugmentation column.

### Metal resistance characterization of the selected bacterial strain

Strain B2A2W2 was tested for W, molybdenum (Mo) and Cu resistance by Minimum Inhibitory Concentration (MIC) determination. A cell suspension was diluted in 5 ml of Phosphate Buffered Saline (PBS) solution containing 8 g NaCl, 0.2 g KCl, 1.44 g Na_2_HPO_4_, and 0.24 g KH_2_PO_4_ per liter pH 7.4 presenting an equivalent OD_600_ of 0.5 in McFarland’s scale. Then, 5 µl of the cell suspension was plated onto a solid R2A medium with increasing concentrations of 3 mM, 5 mM, 10 mM, 20 mM, 50 mM and 100 mM of W and Mo (added as Na_2_WO_4_.2H_2_O (Sigma-Aldrich) and Na_2_MO_4_.2H_2_O (BDH), respectively. In the case of Cu (added as CuCl_2_.2H_2_O (Merck)), the tested concentrations were 1 mM, 2 mM, 3 mM, 5 mM and 10 mM. Bacterial growth was evaluated after 5 days of incubation at 25 ºC.

### Set-up and sampling timeline

**Figure 1:**
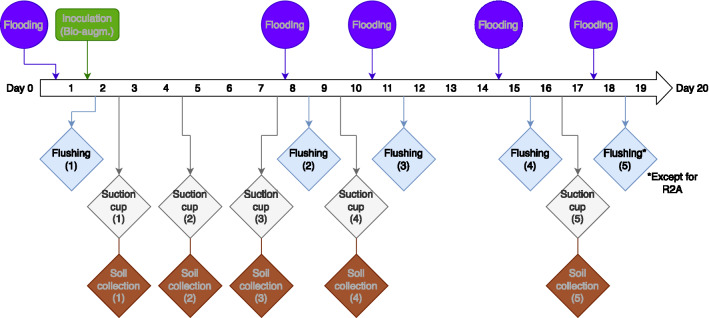
Timeline of the column bioleaching experiment. The systems were flooded on days 1, 8, 11, 15 and 18. The collected samples of the experiment were effluent water (flushing, blue), pore water (suction cup, white) and soil samples (brown).

Two cylindrical columns with 10 cm diameter by 30.5 cm height composed the test system. The columns consisted of a base in ceramic with a net with a 500-mesh grid, a vertical Perspex cylinder and a cover of the same material as the base. The base contained an output valve with a hosepipe for sampling and flooding and a grid on the bottom of the column to prevent the residue from being washed out. Each column was loaded with 3 kg of tailings admixture prepared as described above. After packing, the columns were left overnight at room temperature. The columns were run in parallel under up-flow conditions, ensuring water-saturated conditions. The sequence of events was: on day 1, the bottom inlet was connected to a tank with the growth medium (500 ml) to flood the columns. The system was equipped with a peristaltic pump (KNF-Neuberger Miniport) to allow the eluent to pass through the column (from the bottom to the top), with a flow rate of 1 ml/min, until it reached the top of the column. At this point, the peristaltic pump was stopped and the system was allowed to equilibrate for 24 h^33^. The bioleaching experiment timeline is presented in Fig. 1.

Two different growth media were tested as eluents to activate the autochthonous microbial community as well as to support bioaugmentation. These were R2Ab and modified Mody-Bam-SM (MBM) (containing per liter 10 g mannitol, 1 g glutamate, 0.5 g L-aspargine, 4 g succinate, 1 g ammonium nitrate (NH_4_NO_3_), 0.1 g sodium chloride (NaCl), 0.2 g MgSO_4_.7H_2_O, 0.3 g KH_2_PO_4_, 2 g yeast extract, 20 ml 50% glucose and pH adjusted to 6.0). The two selected growth media differ in carbon source composition and both have shown potential for promoting bioleaching in preliminary batch-scale assays (data not shown). On day 2, immediately before allowing the medium to flow through the column by gravity, 100 ml of bacterial strain B2A2W2 suspension with an OD_600_ of 0.1 was added to the top of one column, as a bioaugmentation strategy. Simultaneously, for the second column (biostimulation column), the same volume of one of the two media was added to the top of the column. On the same day, the bottom plug of the columns were opened, enabling the collection of effluent samples by allowing water to flow through the columns (percolation water) inducing water unsaturated conditions and partial aeration of the columns.

Pore-water samples (i.e. water present held against gravity by the capillary forces of the water unsaturated tailings admixture) were collected with suction cups (Rhizons sampler 2.5 mm, Rhizosphere) by applying negative pressure (-280 hPa). Residue samples were collected for XRF analysis from the middle of both columns, using a Pasteur pipette, without disturbing the system. The column assays were performed over 20 days. Both columns were subjected to repeated flooding and drainage cycles. Flooding was performed with fresh culture medium on days 1, 8, 11, 15 and 18, and left overnight to saturate. Effluent samples were collected on days 2, 9, 12, 16, and 19, while pore water and residue samples were collected on days 3, 5, 8, 10, and 17. Column tests were performed at room temperature and samples were removed from both columns, in parallel.

Collected effluent water samples were centrifuged at 10,000 rpm for 10 min at 4 °C and the supernatant was recovered for further analysis. The pH was measured in all pore water and effluent water samples (electrode pHenomenal™ pH 1,100L), and metals were determined by ICP-MS. The samples of the treated residue (after bioleaching) were analyzed by XRF spectrometer (Epsilon 3XLE), measuring for 100 s with a current of 1000 µA at a voltage of 50 kV.

### ICP-MS post-measurement compressed calibration

The effluent and pore water metal contents were measured by ICP-MS using a Thermo iCAP-Qc ICP-MS, with external calibration and without internal standard^32^. For all intensity measurements, the kinetic energy discrimination (KED) technique was used^34^. The calibration samples used as external standards were prepared using certified reference materials (CRMs), such as Multielement standard solution for JMHW, Periodic table mix 1 for ICP, Tungsten standard for ICP and REE mix for ICP (Sigma-Aldrich). These external standards consist of a main calibration sample and a set of subsequent dilutions. Measurements included a preliminary sweeping of intensities in scanning mode, followed by a peak-hopping procedure for the selected critical target metals. The data collected was initially treated using the Qtegra™ Intelligent Scientific Data Solution™ software (Thermo Fisher Scientific Inc). As per the usual procedure in the Trace Analysis and Imaging Laboratory at the University of Coimbra (TAIL-UC), from the preliminary intensity scans a file was output containing a data set of measured intensities for all samples and mass channels, named ‘Survey Intensities’. After calibration of the target critical metals, and an estimation of the equipment’s sensitivity for these metals, a semi-quantitative estimate of the concentrations of all measurable elements is output by the software as a file named ‘Survey Concentrations’, based on the Survey Intensities and an internal procedure, which estimates the sensitivity of the equipment for all mass channels. Upon inspection of the data in this file, it was found that even for the targeted critical metals specifically selected for the peak-hopping procedure, values differed by orders of magnitude from those obtained by precise determination. A possibility for the inaccuracy of this semi-quantitative estimate is the lack of an internal standard during the measurements, as well as the large range of mass channels coupled with the comparatively low number of selected analytes for peak-hopping.

**Figure 2:**
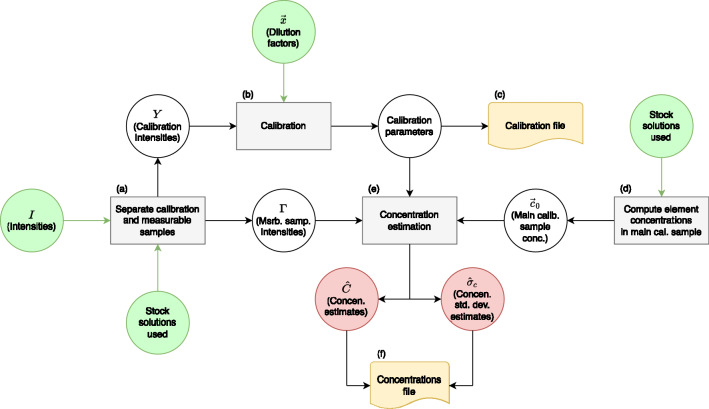
Diagram of the compressed calibration method as implemented in the analysis of bioleachates. Each phase indicated by letters a) to f) is explained in the methods section “ICP-MS post-measurement compressed calibration”.

Considering the relevance of quantifying the concentration of ICs in the measured samples without having to resubmit them for ICP-MS analysis, in the present work a method was developed which could estimate these concentrations based on the Survey Intensities and at the same time provide the degree of precision of the results. The methodology adopted was to use the intensities measured for the external standards to calibrate concentration to intensity relations, following a calibration model with unknown error variance in intensity measurements as a parameter to be estimated. Consequently, the concentrations were estimated based on the intensities measured in the samples through these calibrated relations. A simplified diagram of the method is presented in Fig. 2 and the source code is available at https://gitlab.com/diofalmeida/icpmscc-paper.

The method consisted of (a) separation of the Survey Intensities into intensities pertaining to calibration samples with known concentrations and into intensities pertaining to assay samples with concentrations to be estimated. At this point, information related to elements, which are not present in the external standards, is removed; (b) calibration of relations between the dilution factor of each external standard with respect to the undiluted calibration standard mixture and the related measured intensity, for all possible elements. The calibration model assumes that the error variance in intensity measurements depends on the element but not on the dilution factor. The calibration was performed by considering the intensity and dilution parameters *vs* concentration calibration curve, as well as on the respective estimated variances and covariances; (c) the estimated calibration parameters and related variances and covariance are exported into a Calibration File; (d) the element concentration in the calibration standard mixture is computed according to the critical metals of interest and the external standard preparation procedure; (e) concentrations and related standard deviations are estimated according to the aforementioned calibration parameters and the intensities relating to the samples. This is done by extrapolating from the calibration curve to obtain the dilution factor of each analyte in the sample with respect to the calibration standard mixture and multiplying it by the known concentration of the analyte in the calibration standard mixture; (f) the results are exported into a Concentrations File.

Within the calibration, the variable considered independent was the dilution factor of the external standards (with respect to the calibration standard mixture), as opposed to the direct concentration value. This was done so that the calibration procedure could be the same for all analytes, allowing the process to be vectorized.

Since the calibration was done using external standards and without an internal standard, it is vulnerable to matrix effects and polyatomic ion interferences^32^. We note that the developed method is applied post-measurement, and so does not explicitly account for these interferences. Following the table by May and collaborators^35^, there are some significant possible interferences for the metals quantified and considered for further analysis. However, we believe that the effect of these interferences on our analysis is negligible for three reasons: (1) the use of KED in the intensity measurements reduces the effect of polyatomic ion interferences^34^; (2) since some of the interfering elements originate from the blank solution, these effects are partly corrected during calibration through the use of the background intensity parameter; and (3) the precision of the results is estimated through a standard deviation value which is large enough to yield negligible interference effects.

### Statistical analysis

The ICP-MS measurements of samples from experiments with the R2Ab and MBM media were done on separate occasions. The stock CRMs used to prepare the calibration samples, which were used as external standards differed, and so did the analytes selected for peak-hopping measurements. To compare results between the two media used, the following procedure was followed: (1) the concentration and corresponding standard deviation for all elements present in the external standards were estimated for both media according to the post-measurement calibration method described; (2) results from both media were cross-checked for elements present in the external standards in both cases and the remaining elements were excluded from further analysis; and (3) individual elements’ results were checked for significance in quantification, i.e. elements with estimated standard deviation in the range of estimated concentration were excluded from further analysis. From the surviving elements, those which had also been selected for peak-hopping measurements for both media were chosen for comparing the results of the post-measurement compressed calibration method with those from the standard evaluation done by the Qtegra™ Intelligent Scientific Data Solution™ software used for the peak-hopping procedure, namely the elements Al, Cu and Zn. The validation attempt of the compressed calibration method was done in two steps. First, a visual comparison of the concentration values obtained by the two methods, with error bars representing estimated standard deviations in the case of the CC method, for all samples analyzed. Then, the difference between the concentrations given by both methods was divided by the aforementioned standard deviation for each sample. We could see that the difference between the concentrations given by both methods is within the typical critical value of three standard deviations, for the three analytes under inspection. Further details can be found in Supplementary Figures S1 and S2.

## Results

### Interfering components considered for concentration calculation after compressed calibration

Both effluent (percolation) and pore water samples were collected according to the scheme presented in Fig. 1, to follow the metals mobilization by bacteria and were analysed by ICP-MS. The quantification of the elements defined beforehand was done. After a first analysis, the preliminary ICP-MS intensity measurements obtained were used to be included in the mathematical model here developed, allowing for the quantification of elements not considered for quantification beforehand. Therefore, the IC considered after the procedure mentioned in the Statistical analysis section were Mg, Si, Al, potassium (K), Mn, Fe, Cu, Zn and selenium (Se). Of these, only the elements Al, Cu and Zn had been selected for peak-hopping measurements for both media, and Mn had only been selected for MBM medium. Comparisons between the post-measurement method and the standard ICP-MS procedure were made for Al, Cu and Zn, and were in agreement. Further details and graphical representations of this comparison are available in the supplementary material (Supplementary Figures S1 and S2). For the remainder of the conducted study, only the results of the post-measurement calibration method were used.

### Effect of medium composition and effect of biostimulation or bioaugmentation on leaching of elements

Both media, R2Ab and MBM, were used to activate the microbiota and to support bioaugmentation, separately. The bacterial strain B2A2W2 was the one selected for bioaugmentation conditions since it showed high resistance to different metals, namely Cu, W and Mo (Supplementary Table S3) and ability to produce siderophores. Genome analysis of the strain indicated the presence of organic acids extrusion and production pathways. Additionally, preliminary batch assays revealed ability of strain B2A2W2 to bioleach the mine residues (data not shown).

The concentration of IC in the samples differed over time, in most cases. The average concentrations obtained from both media were calculated directly from the corresponding Survey Intensities and subsequently translated via the calibration curves to concentration values (Supplementary Fig. 3).

**Figure 3:**
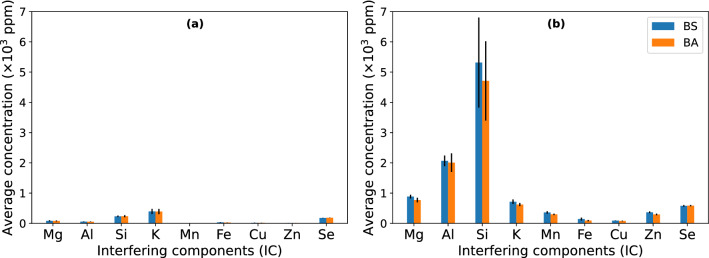
Time-averaged concentrations of selected IC in effluent water samples from MBM (**a**) and R2Ab (**b**) media. Biostimulation (BS) with the culture medium is represented in blue and bioaugmentation (BA) with strain B2A2W2 is represented in orange. The y-axis scale was set to the same range for a clear comparison between both media tested. Error bars correspond to estimated standard deviations.

Generally, with the R2Ab medium, mobilization of metals from Panasqueira mine tailings was higher than with the MBM medium. This increased mobilization was of approximately 20 times for Si (from 233 ± 26 ppm to 5315 ± 1487 ppm), 40 times for Al (from 55 ± 7 ppm to 2065 ± 175 ppm) and 10 times for Mg (from 84 ± 18 ppm to 884 ± 62 ppm) – values shown are for the biostimulated sediments, similar values were obtained in the bioaugmented case. The metals Mn and Zn were only bioleached in detectable concentrations when the R2Ab medium was used. The average concentration of all leached elements for the R2Ab medium assay showed small differences between biostimulation and bioaugmentation but, even for Si, were not significant (Fig. 3).

The pH of the assays was acidic (varied between 2.5 and 3.5), stable during both experiments and no high variations were observed between pore and effluent water. The pH was 0.5 to 1 value higher when the system was flooded with R2Ab medium. Taking into consideration the concentrations of the metals leached and the fact that the pH was stable at a value not inhibitory to the strain used in biostimulation, the R2Ab medium was selected for further experiments (Supplementary Figure S3).

### Bioleaching of metals as assessed from column effluents and pore water samples

**Figure 4:**
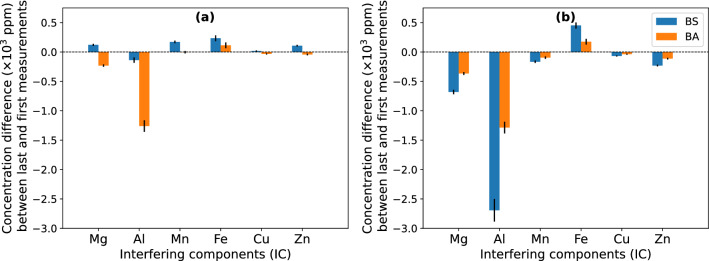
Absolute time-dependent variation of concentrations between last and first measurements during the assay with R2Ab medium for the elements Mg, Al, Mn, Fe, Cu and Zn, determined in effluent water (**a**) and pore water (**b**) samples, from both biostimulation (BS) and bioaugmentation (BA) conditions (represented in blue and orange, respectively) Error bars correspond to estimated standard deviations.

The results shown in Fig. 4 illustrate the absolute time-dependent variation of the concentration of non-target ICs in the leachates, between the last (day 20) and the first (day 2) measurements, when the R2Ab medium was used to biostimulate or bioaugment the residues. When applying bioaugmentation with strain B2A2W2, the metal concentration in the effluents showed a negative variation for the elements Mg, Al, Cu and Zn as a consequence of a decrease in concentration after 20 days of incubation. However, the element Fe showed a positive variation since effluent concentrations increased over time. With biostimulation, the temporal variation of metal concentrations in effluent water was positive except for Al. Additionally, when considering the metal concentrations in the pore water, the tendency of the variation of the elements Al and Fe was the same. On the other hand, the concentration of Mg, Mn, Cu, and Zn decreased, both under biostimulation and under bioaugmentation in pore water.

**Figure 5:**
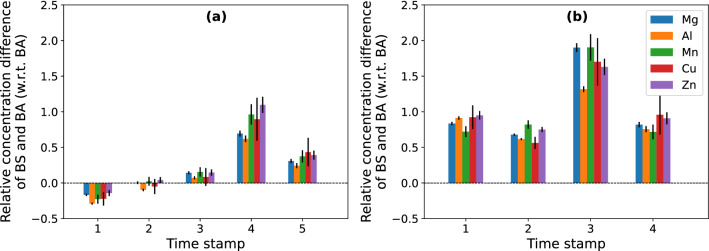
The relative deviation between concentrations in samples obtained by biostimulation (BS) with R2Ab medium and bioaugmentation (BA) with strain B2A2W2, for the elements Mg, Al, Mn, Cu and Zn (represented in blue, orange, green, red and purple, respectively), in effluent water (**a**) and pore water (**b**) samples. Error bars correspond to estimated standard deviations.

For each sampling time, the difference in IC concentrations in samples, obtained under biostimulation and bioaugmentation conditions with R2Ab medium, was determined (Fig. 5). In the column effluents, all quantified elements showed the same tendency. Higher concentrations were observed under bioaugmentation conditions at times 1 and 2, but after time 3 (i.e. 10 days of incubation) effluent concentrations were higher under biostimulation conditions. On the other hand, pore water concentrations under biostimulation were higher at all times.

The highest metals concentration was detected at timestamp 3. This holds irrespective of the leaching methodology (biostimulation or bioaugmentation) or the sampling technique (effluent collection or pore water collection).

### Time course of ICs obtained with R2Ab bioleaching

**Figure 6:**
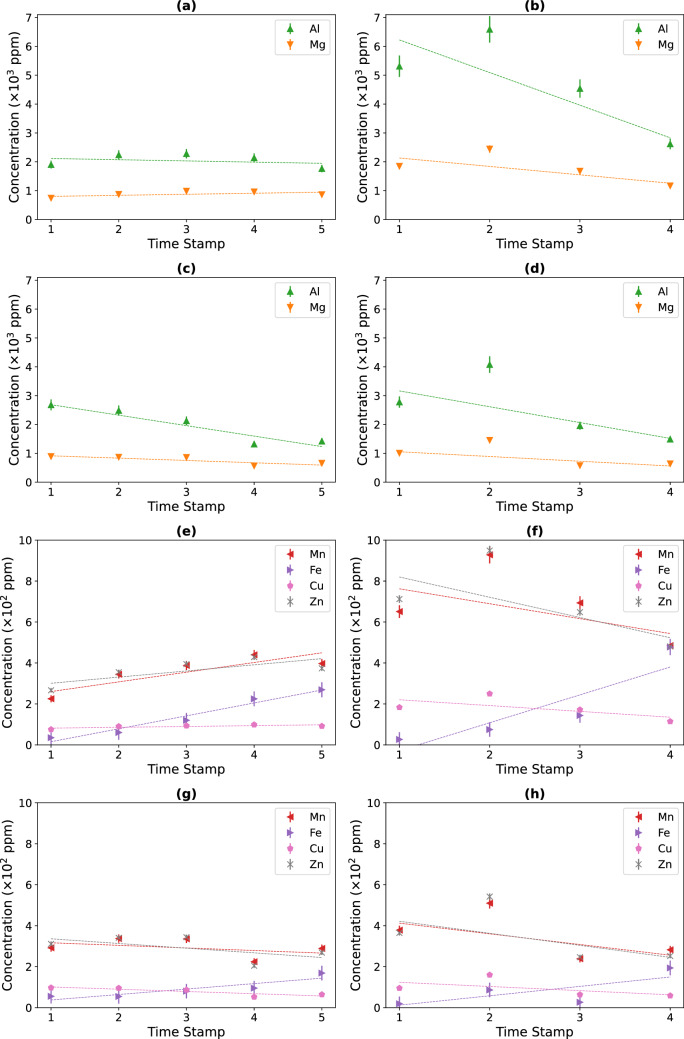
Temporal variation of the IC: Al and Mg (**a**–**d**), and Mn, Fe, Cu and Zn (**e**–**h**). Leached concentration is represented in green, orange, red, purple, lilac and grey, respectively. The overtime variation of the leached IC concentrations is represented for biostimulation (**a**, **b**, **e** and **f**) and bioaugmentation (**c**, **d**, **g** and **h**) conditions, effluent water (**a**, **c**, **e** and **g**) and pore water (**b**, **d**, **f** and **h**) samples (means ± standard deviations-error bars-from each measurement).

The time course of leached non-target IC elements Al, Mg, Mn, Fe, Cu and Zn concentration was followed. Figure 6 shows that under biostimulation conditions the Al, Mg, Mn, and Zn effluent concentrations were less than half of the respective pore water concentrations (Al 2,000 vs 6,000 ppm; Mn and Zn 200 vs 800 ppm). However, this was not observed under bioaugmentation conditions. In general, metals mobilization was more pronounced under biostimulation and in pore water at the beginning of the assay. At the end of the experiment, the IC concentration in pore water tended to equilibrate with percolation water samples. The element Al was the major IC leached from the residues. High concentrations of this element were detected in the pore water under biostimulation conditions. The Al concentration in pore water samples reached 6,000 ppm but decreased with time (Fig. 6), showing a negative time-dependent variation. In the case of the element Fe, this element presented a time-dependent positive relation since leachate concentration increased with incubation time. This effect was more visible in pore water when the system was activated by biostimulation. The Fe concentration increased from 2 to 400 ppm during the incubation time. When comparing effluent water, Fe leaching was more effective under biostimulation conditions (Fig. 6). Since repeated flushing eventually leads to the draining of pore water Fe effluent concentrations can be expected to increase over time. The elements Zn and Mn showed a similar bioleaching profile for pore water, with metal concentrations decreasing over time. The effluent level of both metals increased under biostimulation conditions. On the other hand, during bioaugmentation, both metals showed similar and rather constant concentrations in the effluent and pore water (Fig. 6). Critical metals, such as lithium (Li), cobalt (Co) and cadmium (Cd), were also detected in pore and effluent water samples, but in low concentrations comparable to their estimated standard deviations.

### Elemental composition of the bioleached solid residue

**Figure 7:**
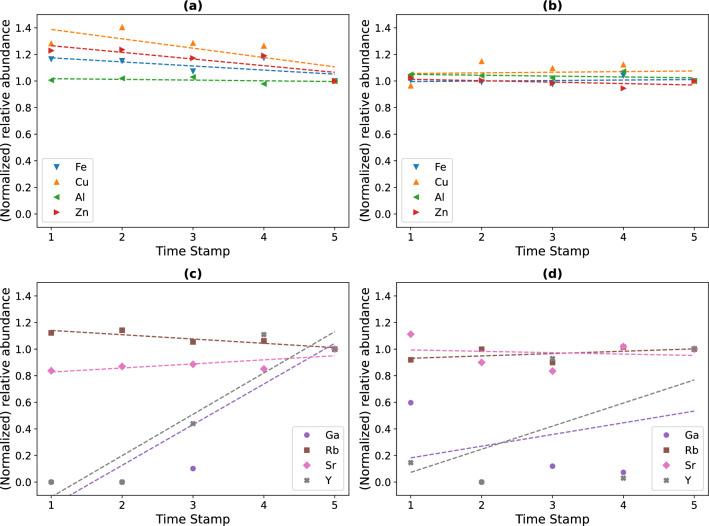
Results of sediment composition analysis by XRF. Relative abundance normalized to the last measurement for the five time-stamped soil samples, for both biostimulation (**a** and **c**) and bioaugmentation (**b** and **d**) conditions. The last measurement was used for normalization because the first measurement was below the detection limit of the equipment for the metal Ga in the biostimulated sediment. The relative abundance (normalized to the last measurement) is shown for non-target IC, namely Fe, Cu, Al and Zn, represented in blue, orange, green and red, respectively (**a** and **b**). The critical target metals Ga, Rb, Sr and Y relative abundance is represented in purple, brown, pink and grey, respectively (**c** and **d**).

An evaluation of the relative abundance of detectable metals in the leached mine sediments was done by XRF, after biostimulation of the sediments with R2Ab medium, and after bioaugmentation with strain B2A2W2 in R2Ab medium. After biostimulation, the relative content of the IC elements Cu, Zn and Fe in the treated residue decreased. All relative concentration values were normalized to the last measurement. These normalized relative concentrations shifted slightly from values 1.4 (Cu) or 1.2 (Zn, Fe) to unity (Fig. 7). For bioaugmentation conditions, the decrease in the relative content of the non-target IC was very low.

In the biostimulated residues the relative concentration of the critical target metals yttrium (Y) and gallium (Ga) increased up to 10 times compared to the initial concentration (Fig. 7). Also, bioaugmentation with strain B2A2W2 lead to the relative increase of Y and Ga content of the residue, yet at a much lower level compared to biostimulation. Neither biostimulation nor bioaugmentation of residues led to a relative increase of rubidium (Rb) or strontium (Sr) in the bioleached sediments. The failure to detect an increase of the elements poorly represented in the sediments could be related to the presence of new elements that could appear, for example, due to biomass increase affecting the mass balance.

## Discussion

Bioleaching can lead to the enrichment of target metals in the leachate or alternatively, to their enrichment in the solid residue when ICs are preferentially mobilized^36^. To follow the ICs mobilization, a modified calibration model was developed *in domo*. The model makes use of the preliminary ICP-MS intensity measurements and allows for the quantification of elements that were not considered for quantification beforehand. This was used to follow the non-target leached ICs that compose the Panasqueira mine tailings.

In the present work, metals bioleaching from mine tailings was studied in a column leaching experiment by analyzing both effluent (percolation) and pore water samples. This approach is particularly suited for the upscaling of systems^15^. The microbial community composition of the Panasqueira mine tailings was described and strains were isolated for further studies^13^. In previous works, the isolates from this mine demonstrated the capacity to resist and interact with metals^37^. Strategies such as selective metal accumulation through membrane transporters, the production of metal-chelating biopolymers and the production of siderophores for selective metal capture were identified in strains of the genera that compose the microbiome of the Panasqueira mine^38^. To activate this community metabolically, we used two culture media with different carbon source compositions that had successfully promoted bioleaching in previous batch-type assays. In the column experiments, the R2Ab medium promoted more overall metals mobilization from Panasqueira mine tailings than the MBM medium, confirming that the culture medium is a key factor in bioleaching assays^39^. Nevertheless, none of the culture media seemed to promote bioleaching by bioaugmentation with *D. polyhydroxybutyrativorans* strain B2A2W2, although it was able to leach these sediments when incubated in batch shaking flasks (data not shown). Moreover, since this strain is resistant to several metals, it is expected to be biologically active in the presence of a mixed solution of metals and metalloids, at high concentrations. Despite the pH of the residues in the tailings basin varying between 6 and 7^13^, during the assays, the pH was acidic, between 2.5 and 3.5, with similar values in pore water and effluent samples. This decrease in the pH may be related to the potential for low net acid generation potential detected in the residues. The composition of the sediments includes low content of pyrites and its biological oxidation may lead to acidification and mobilization of metal species during incubation^40^.

Different works showed differences in elements’ concentrations between pore and effluent water samples due to diffusion processes^41^. In this work, we followed the temporal variation of the leached concentration of the ICs as Al, Mg, Mn, Fe, Cu and Zn, in pore water and effluent samples. This variation considered the IC removal from the residue relative to the increase of critical target metals on the tailings. Concentrations determined by post-measurement compressed calibration are not as precise as those obtained by the standard peak-hopping calibration and measurement method. However, for analytes with sample concentrations above their concentration in the main calibration standard mixture, the results are precise, with lower relative standard deviations than would be expected given the lack of precision in the Survey Intensities file data relative to the peak-hopping procedure data. The method thus allows us to draw conclusions on a greater number of analytes in the samples than considered in the calibration standard. This gives us detailed information on the variation of the composition of the measured samples if the analysis is complemented with other techniques such as XRF^42,43^. Additionally, if the calibration set contains a wide range of elements, this method offers a sound and more rigorous alternative to the semi-quantitative estimation provided of the concentrations generated by the Qtegra™ software (Survey Concentrations) for a larger number of analytes. It also provides a precision estimate (standard deviation of each measurement) and does not require additional curve fitting, since the sensitivities of the measurable elements (present in the calibration standard mixture) can be estimated directly from the calibration. Finally, as a post-measurement method, it allows re-evaluating data from previous measurements and possibly drawing new conclusions from the Survey Intensities files. The post-measurement compressed calibration for the quantification of metals based on ICP-MS allowed us to observe that under biostimulation conditions ICs were preferentially released into pore water at the beginning of the assay. This is consistent with the pore water micrometric scale since bacteria interact with the solid phase to bioleach the metals^25^. On the other hand, at the end of the experiment, the concentration of metals in the pore water and effluent samples showed similar values, suggesting that the column system tends to an equilibrium between pore and effluent water after 23 days of incubation as described^27^.

Panasqueira is a wolframite deposit and contains associated granites with alterations that are prone to yield rare metals to the coeval quartz-wolframite veins^44^ so the leached elements and the increase in critical metals Ga and Y were expected. The element Al was the IC leached in the highest concentrations. It reached a maximum of around 6,000 ppm in the pore water after 5 days of incubation, when biostimulation conditions were applied. Under the same conditions, the elements Cu and Zn were found also in high concentrations in the pore water reaching 300 and 500 ppm, respectively. Al is a major element and is expected to come from the feldspar microliths in the quartz and granite or the micas of the ore deposit^44,45^. As opposed to the other elements, the Fe concentration increased steadily over time. This suggests that increasing incubation time will lead to the mobilization of Fe and probably other elements from the residue, related to the production of ion Fe^2+^ species^46,47^. The Fe mobilization observed at late incubation may be related to the presence of low numbers of slow-growing members of the genera *Thiobacillus* and *Acidithiobacillus* that can oxidize ferrous and sulfur compounds and play an important role in Fe and S cycling^46,47^.

In consistency with the appearance of these metals in the obtained leachates, the XRF evaluation of the leached residues showed a percentage decrease of the IC as Zn, Cu, and Fe. The expected decrease of the relative concentration of Al in the residue was not visible due to its large concentration compared with a relatively small variation. In consequence, and since critical metals were below quantification limits in the leachates, a relative increase of critical target metals that compose the residue, such as Ga and Y, was obtained in the biostimulation residue. Thus, it was proved that the biostimulation condition of the tailings from Panasqueira mine Basin 1, with R2Ab medium, can be used as a strategy to promote the removal of interfering metals from tailings by leaching them out, consequently increasing the relative abundance of critical metals in the sediment. This work also suggested that we could take advantage of the high microbiological diversity existing in the respective basin^13^ and biostimulate it to promote effective bioleaching. Although bioaugmentation in presence of the autochthonous bacterial strain did not improve the tailings microbiome bioleaching activity, in basin areas with lower microbiological diversity, the bioaugmentation with the strain B2A2W2 may enhance microbiological activity and metal mobilization.

To summarize, biostimulation of the Panasqueira mine residue microbiota resulted in the efficient bioleaching of IC (non-target metals). By contrast, the bioaugmentation with strain B2A2W2 did not improve bioleaching. The R2Ab medium provided a supplementation with diverse low-level carbon sources and efficiently promoted the bioleaching of non-target IC. This suggests that the medium is an efficient way to biostimulate the microbiota of Panasqueira mine tailings. Our results indicate that residues enriched with high-value critical target metals can be obtained by biostimulating the residues and removing the IC.

## Supplementary Information

Supplementary Information 1.

Supplementary Information 2.

Supplementary Information 3.

Supplementary Information 4.

## Data Availability

The whole 16S rRNA sequencing data of the strain has been deposited on the Genbank repository under accession number (OK644232). The strain used in this study *Diaphorobacter polyhydroxybutyrativorans* B2A2W2 was deposited in the UCCCB culture collection under the identifier UCCCB 148. The data collected by ICP-MS referenced in this paper and the source code used to perform the compressed calibration have been made available in the GitLab repository https://gitlab.com/diofalmeida/icpmscc-paper.
