# Supplementary figures and images for: Post-measurement compressed calibration for ICP-MS-based metal quantification in mine residues bioleaching
Source: Sci Rep. 2022 Sep 26;12:16007. doi: 10.1038/s41598-022-19620-8 (PMC9512927; doi:10.1038/s41598-022-19620-8)

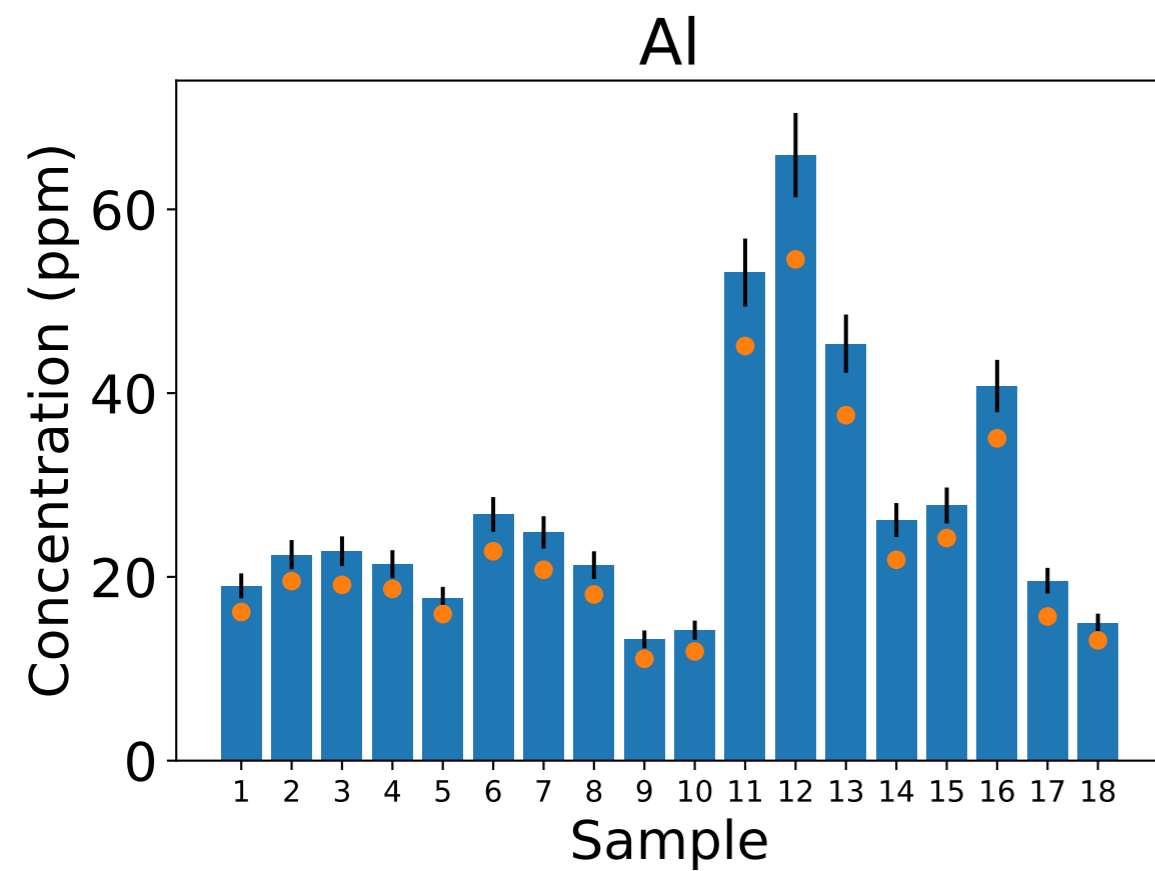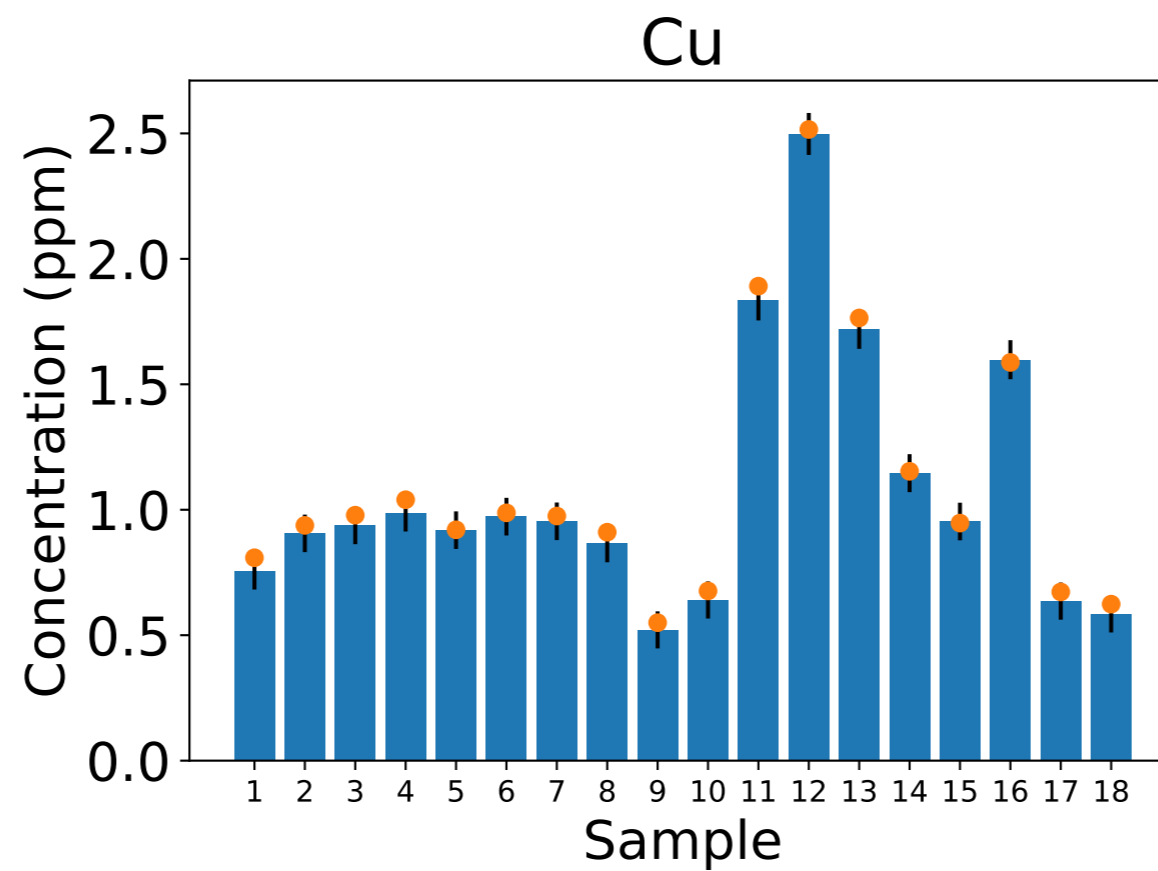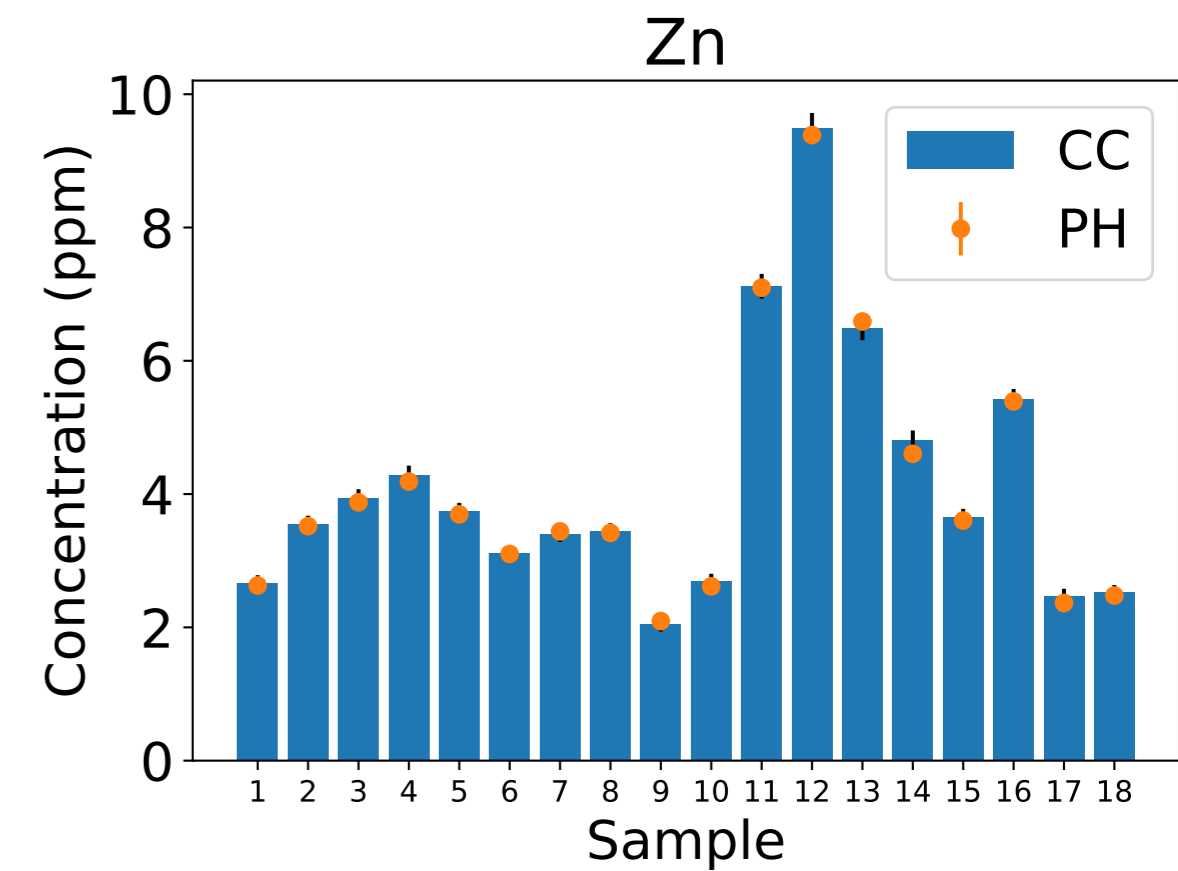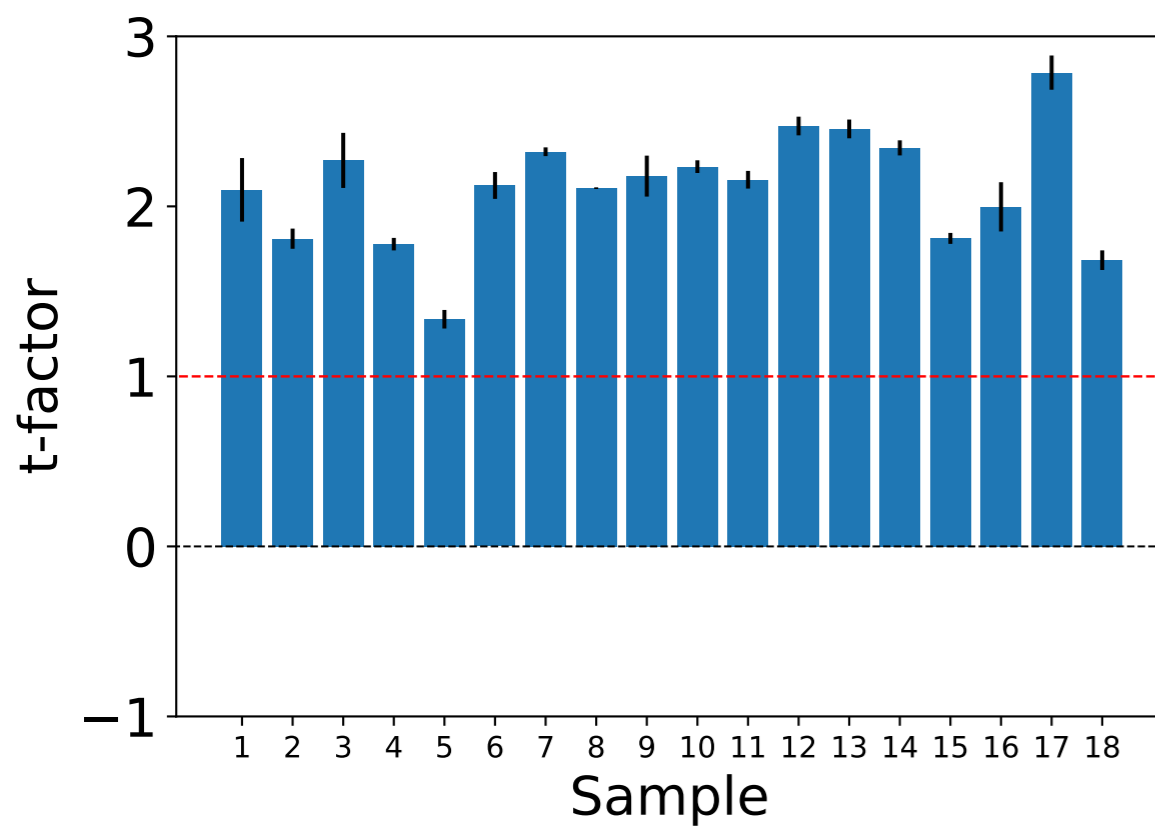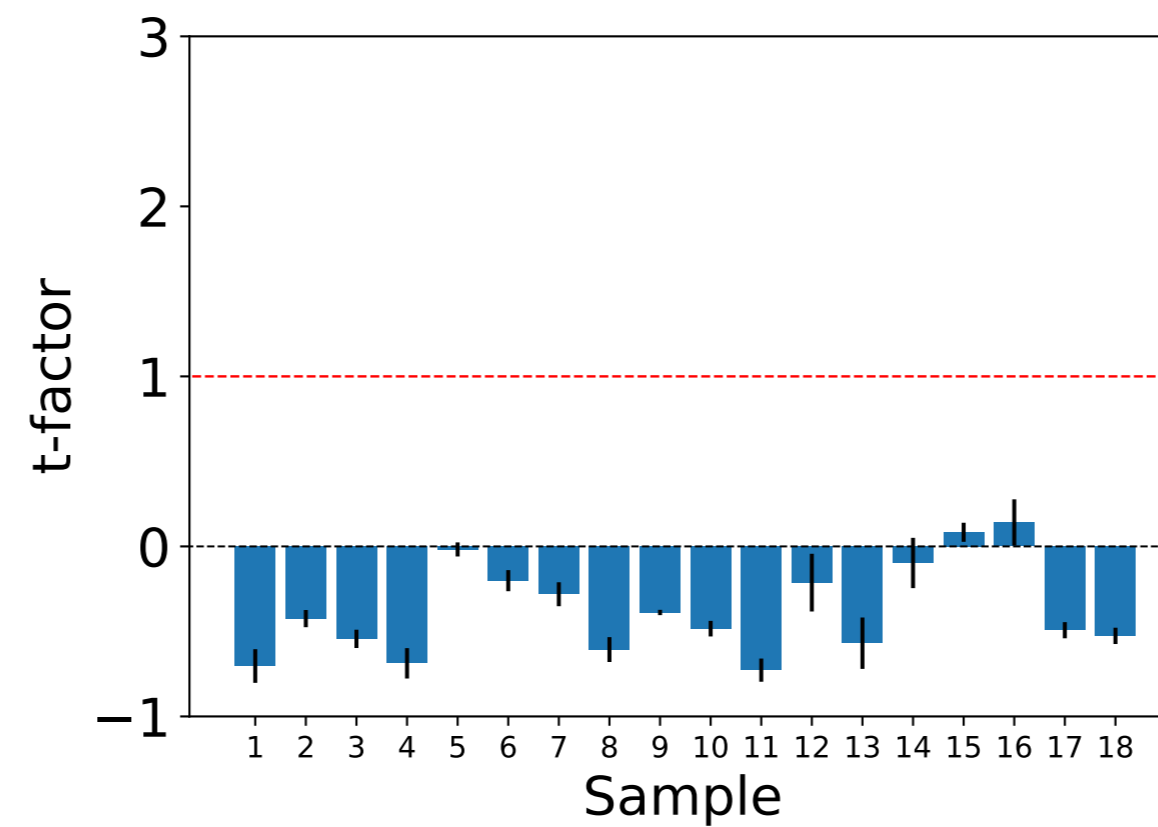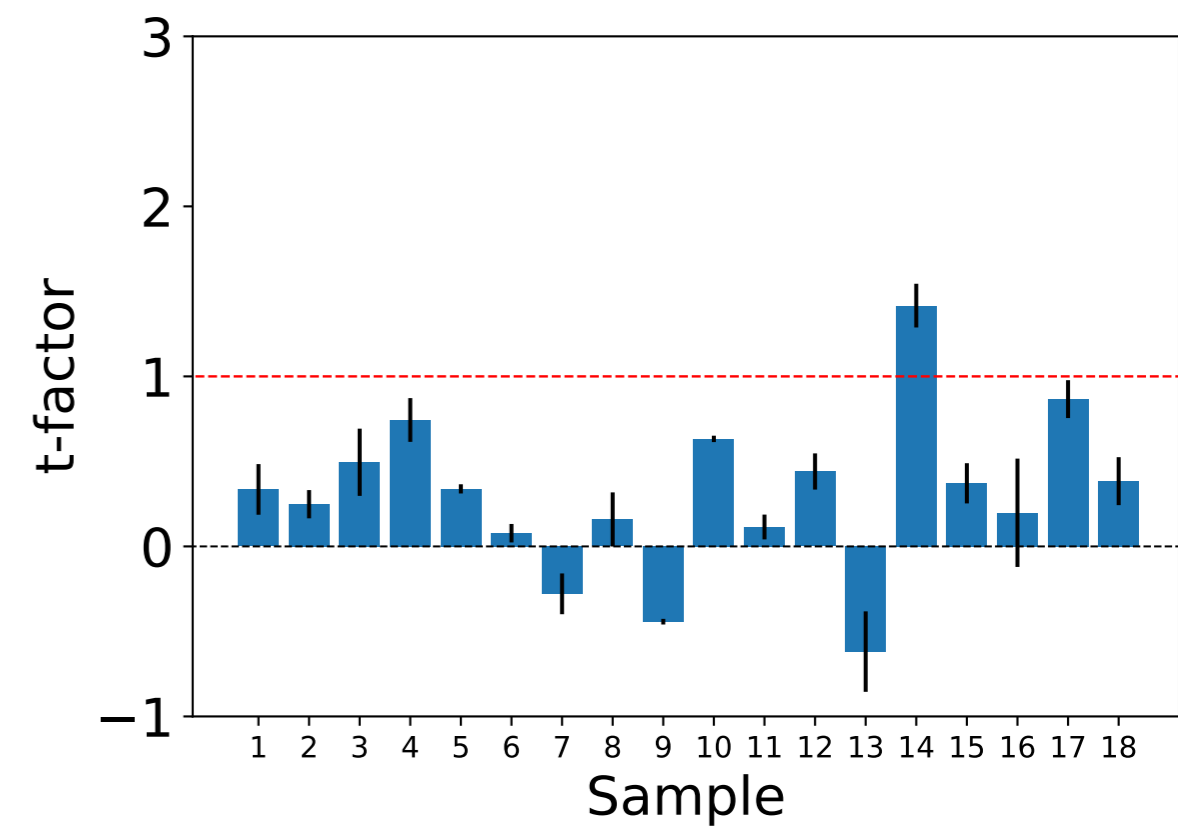

Supplement: Supplementary file 1 — Supplementary Information 1. [file 41598_2022_19620_MOESM1_ESM.pdf]

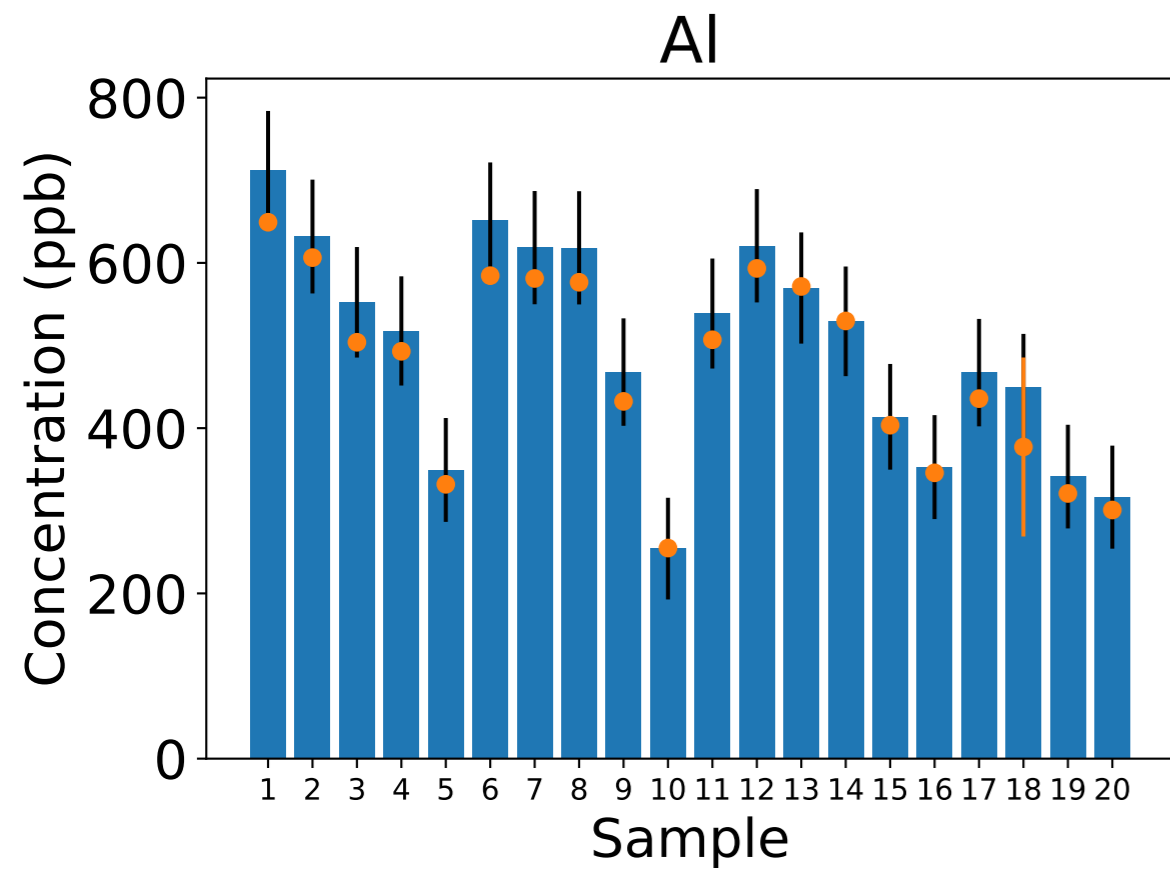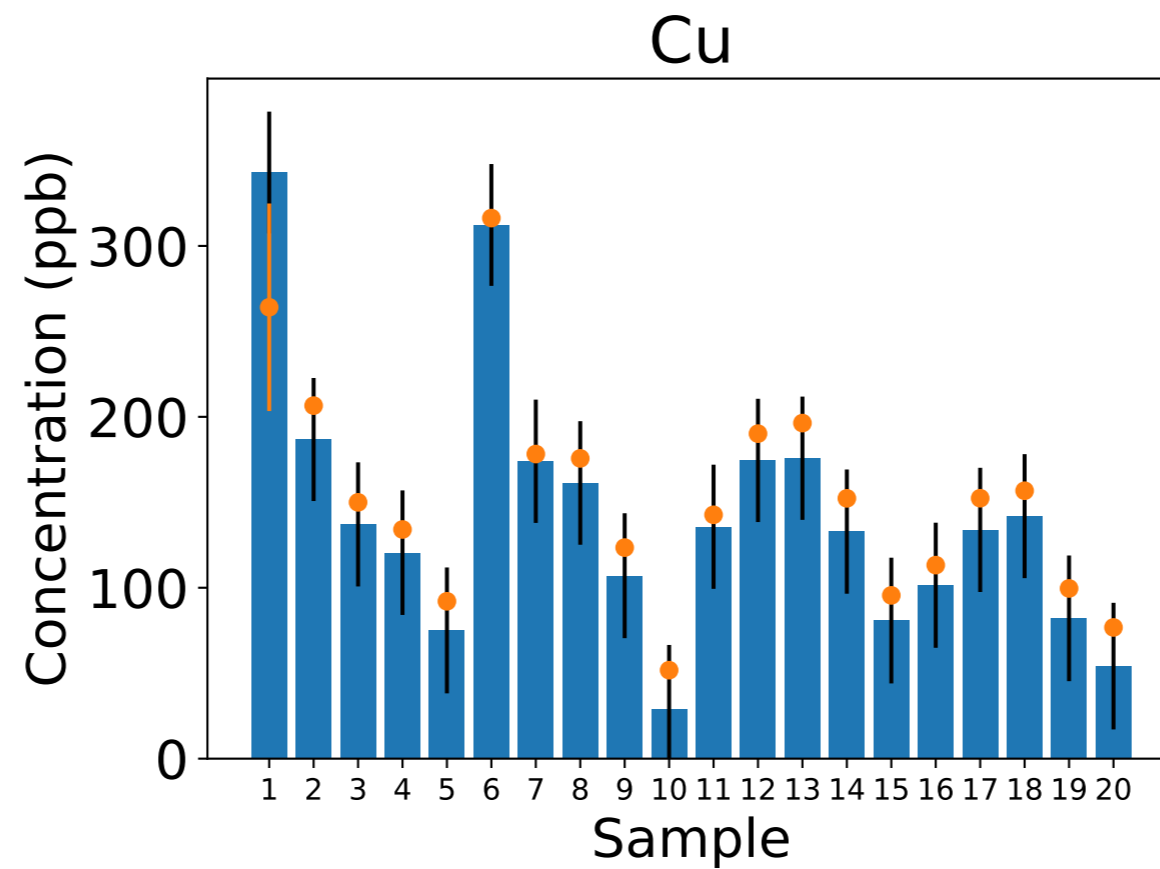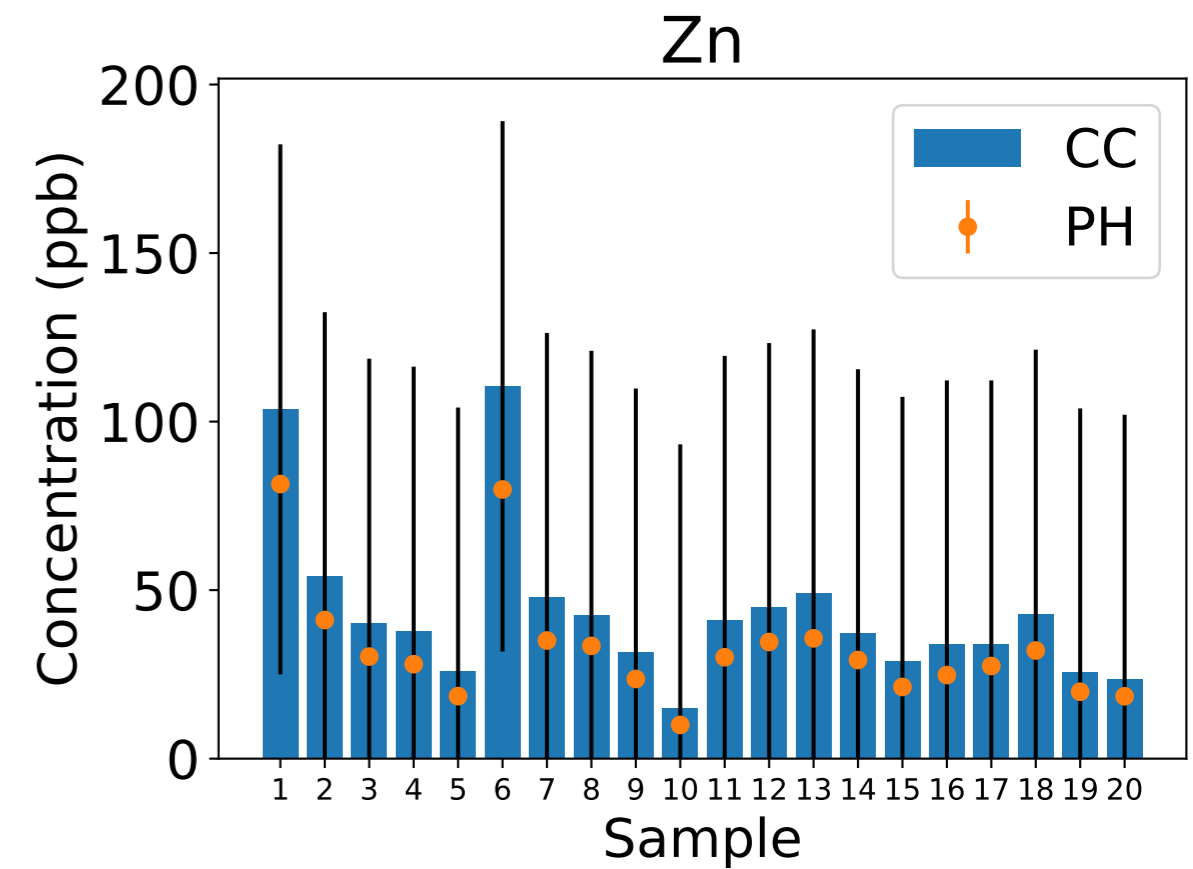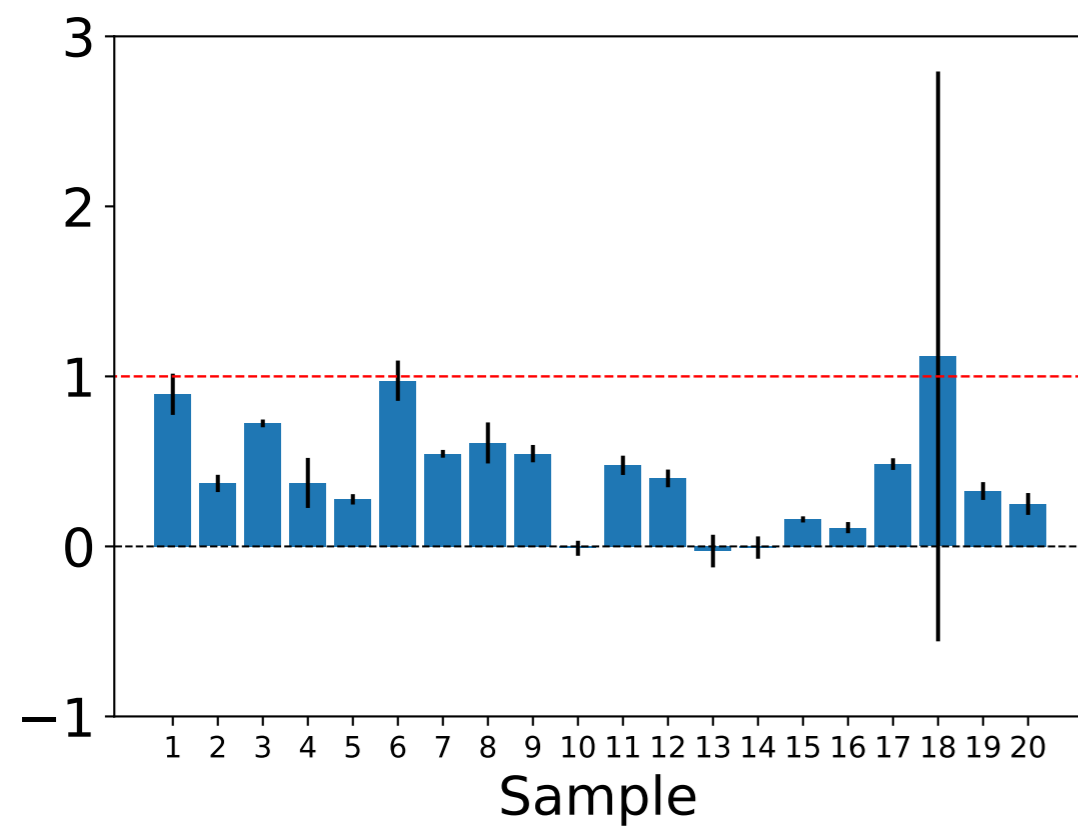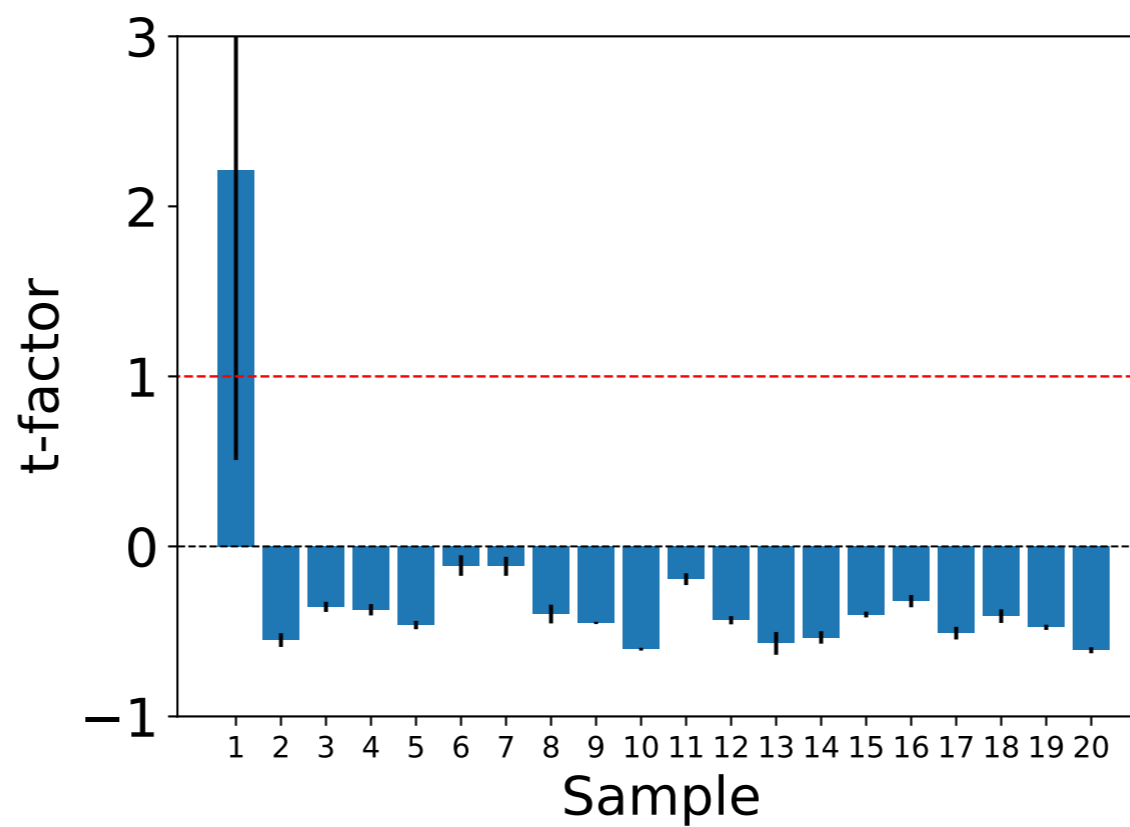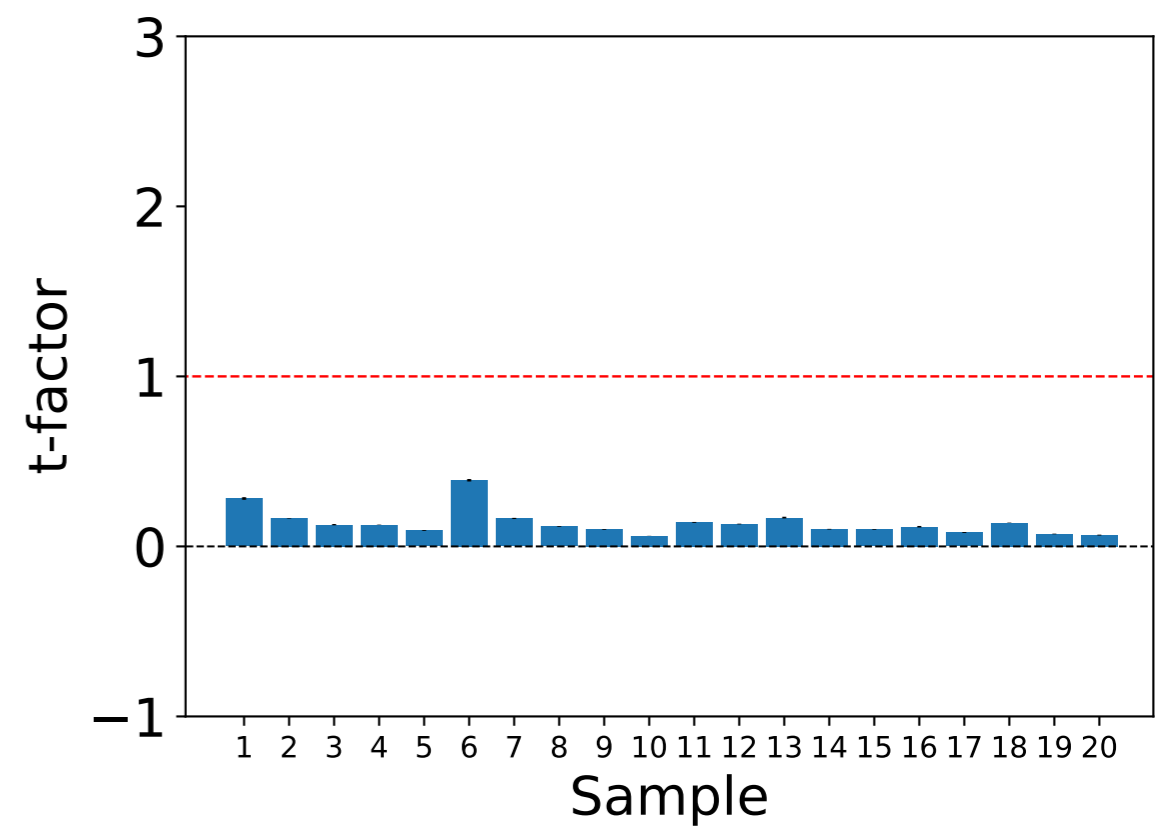

Supplement: Supplementary file 2 — Supplementary Information 2. [file 41598_2022_19620_MOESM2_ESM.pdf]

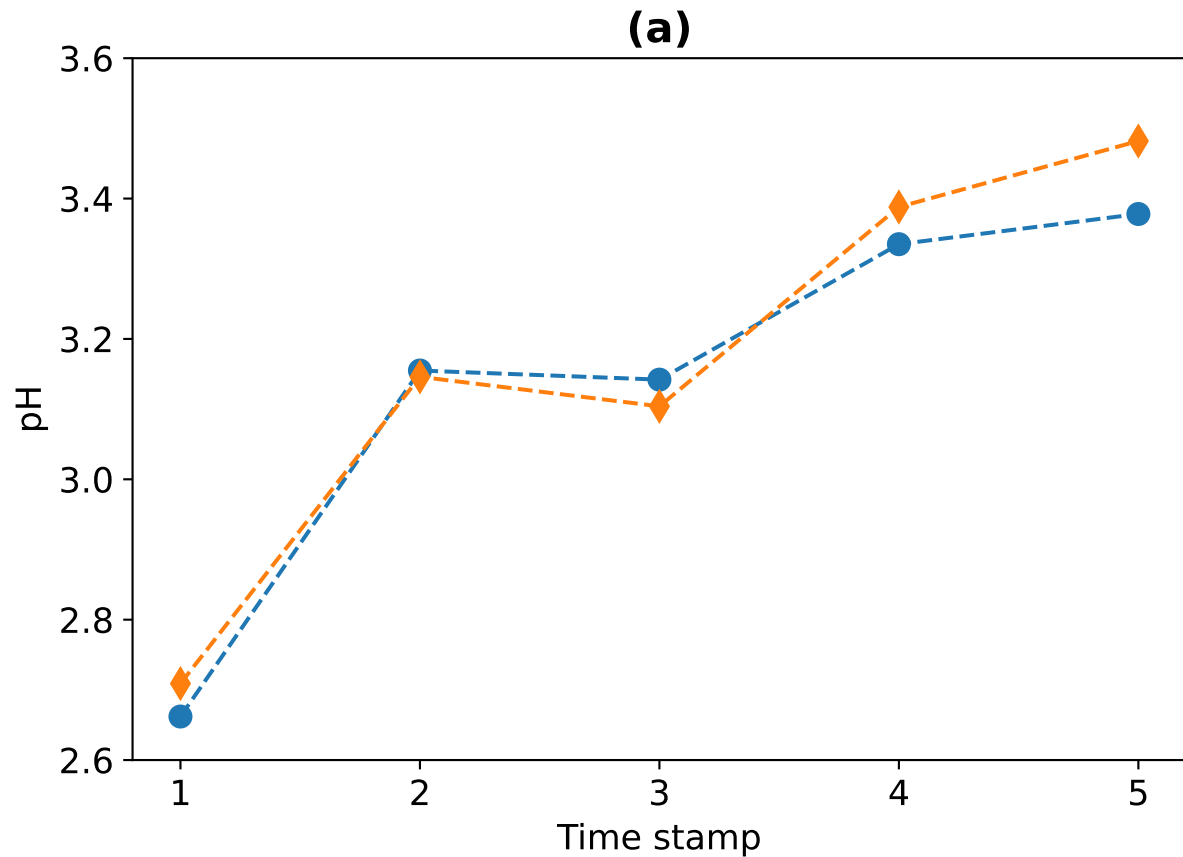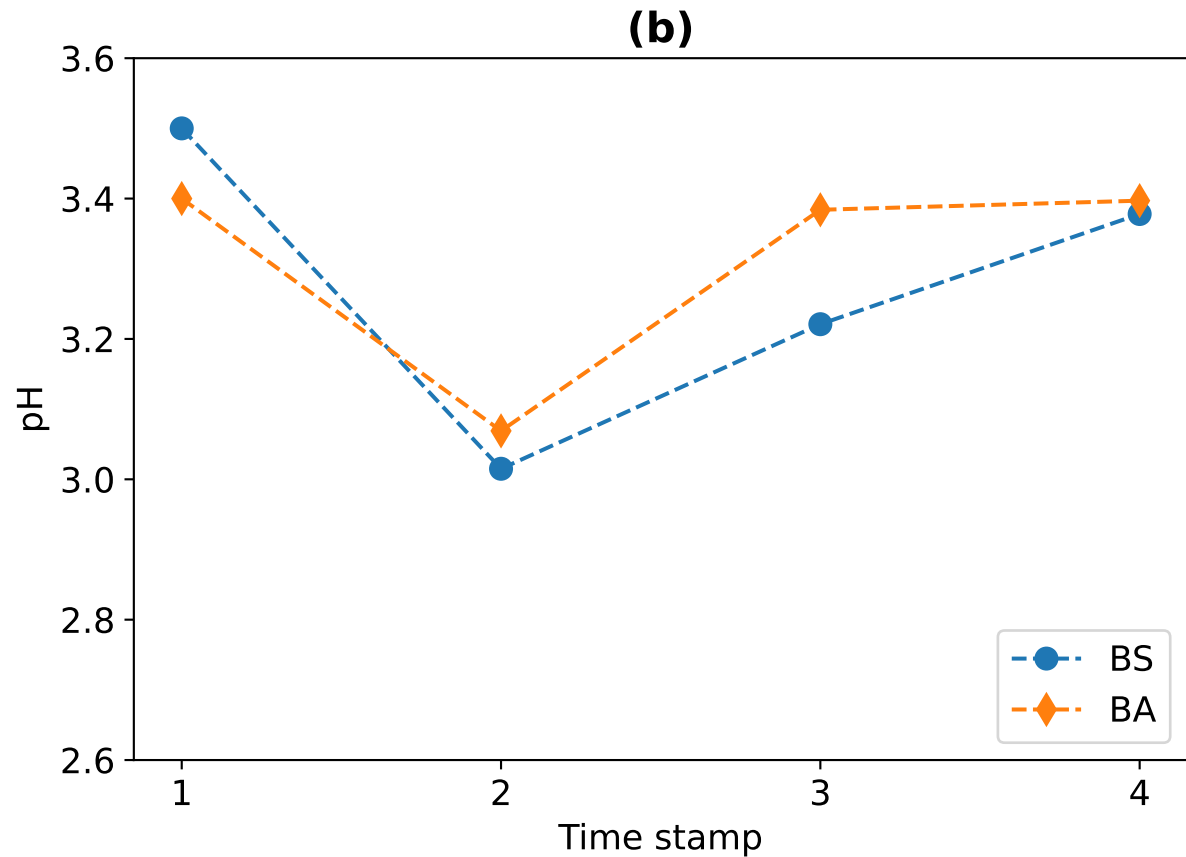

Supplement: Supplementary file 3 — Supplementary Information 3. [file 41598_2022_19620_MOESM3_ESM.pdf]
