# Supplementary material for: Post-measurement compressed calibration for ICP-MS-based metal quantification in mine residues bioleaching
Source: Sci Rep. 2022 Sep 26;12:16007. doi: 10.1038/s41598-022-19620-8 (PMC9512927; doi:10.1038/s41598-022-19620-8)
Supplement: Supplementary file 4 — Supplementary Information 4. [file 41598_2022_19620_MOESM4_ESM.pdf]

Supplementary Material

Post-measurement compressed calibration for ICP-MS-based metal quantification: a case study of dynamic columns bioleaching of Panasqueira mine residues

Beatriz Rito[1#], Diogo Almeida[1#], Carina Coimbra[1], Diogo Vicente[1], Romeu Francisco[1], Rita Branco[1], Harald Weigand[2] and Paula Vasconcelos Morais[1,*]

Supplementary Table S1. Metals composition of Panasqueira mine residues from Basin 1 (B1) and Basin 2 (B2) (adapted from Chung, Ana Paula, *et al*. 2019).

Chemical Elements (ppm)	Basin 1 Average (ppm)	Basin 2 Average (ppm)
**F**	8020.0	7793.3
**Na**	6155.0	4928.4
**Al**	105135.5	83730.9
**Si**	273963.5	269066.3
**P**	1371.8	1607.5
**S**	6757.1	18140.8
**K**	41394.1	30737.7
**Ca**	3490.1	4979.1
**Ti**	8373.3	6790.0
**Fe**	54947.2	69472.1
**Sc**	15.0	11.9
**V**	153.3	104.0
**Cr**	112.9	89.9
**Mn**	834.2	830.6
**Co**	17.9	21.9
**Ni**	61.8	66,5
**Cu**	1960.6	2892.1
**Zn**	4431.5	8607.6
**Ga**	29.3	22.2
**Ge**	14.6	7.9
**As**	3079.1	22397.6
**Rb**	578.4	396.2
**Sr**	75.3	73.1
**Y**	27.3	19.5
**Zr**	197.1	176.0
**Nb**	13.8	10.2
**Mo**	2.8	4.0
**Ag**	15.1	16.7
**Cd**	50.8	103.4
**Sn**	310.2	408.5
**Sb**	3.3	9.4
**Cs**	84.0	37.7
**Ba**	523.7	361.9
**La**	31.0	21.0
**Ce**	110.9	307.6
**W**	1461.1	1155.8
**Pb**	108.5	114.0

Supplementary Table S2. Panasqueira mine residues' metals composition (adapted from Chung, Ana Paula, *et al*. 2019).

D90 (µm)	BOD (g/L)	TOC (%)	pH	NAG (kg H_2SO_4/t)
223.1	0.15	0.65	5.9	7.8
42.7	0.04	0.66	5.9	7.6
66.9	0.54	0.71	6.0	8.0
931.6	0.19	0.89	7.0	16.8
751.3	2.94	0.81	6.9	17.4
554.6	0.01	1.05	6.5	17.0

D90 - Maximum diameter for 90% of the particles, BOD - Biological Oxygen Demand, TOC, Total Organic Carbon in sediments, NAG - Net Acid Gen

Supplementary Table S3. *Diaphorobacter polyhydroxybutyrativorans* strain B2A2W2 tungsten (W), molybdenum (Mo) and copper (Cu) resistance determination on solid R2A medium.

Bacterial strain	Tested metals	Minimum Inhibitory Concentration (MIC) (mM)
B2A2W2	W	100
	Mo	100
	Cu	2

+, positive result; (+), weakly positive result; −, negative result

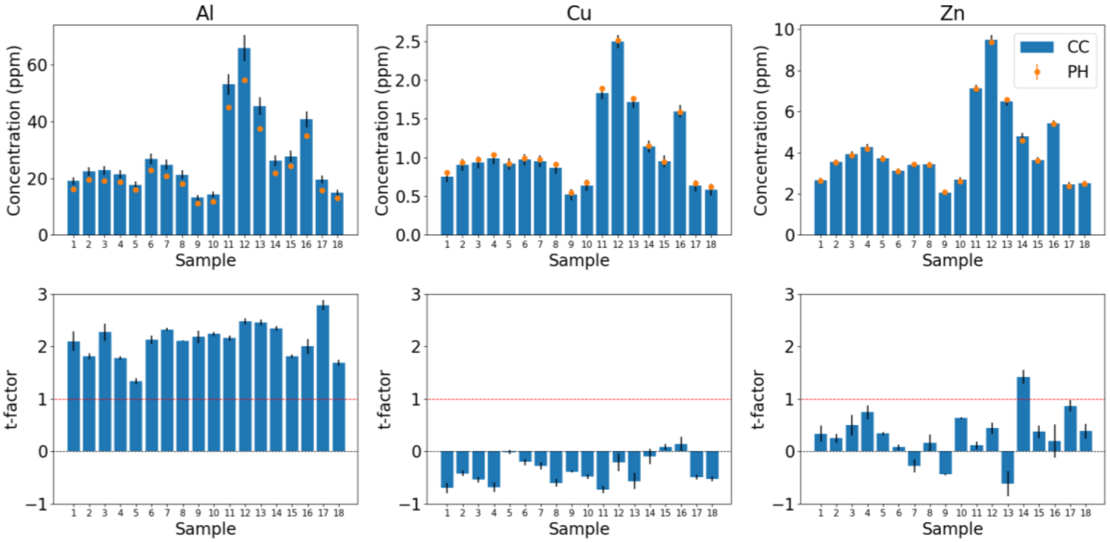

Supplementary Figure S1. Comparison between compressed calibration (CC) and direct ICP-MS peak hopping (PH) results for the concentrations of elements Al, Cu and Zn in the diluted samples directly measured in TAIL-UC, for the R2A medium. In the first row, CC results are shown as blue bars and the corresponding estimated standard deviation in black error bars. The PH results are shown as orange circles, with corresponding estimated standard deviations, although obscured by the circles in most cases. On the second row, blue bars indicate the *t*-factor computed as the difference between the CC and PH estimated concentrations, divided by the corresponding estimated standard deviations for CC. The error bars indicate, in this case, the estimated standard deviations for PH divided by the estimated standard deviations for CC.

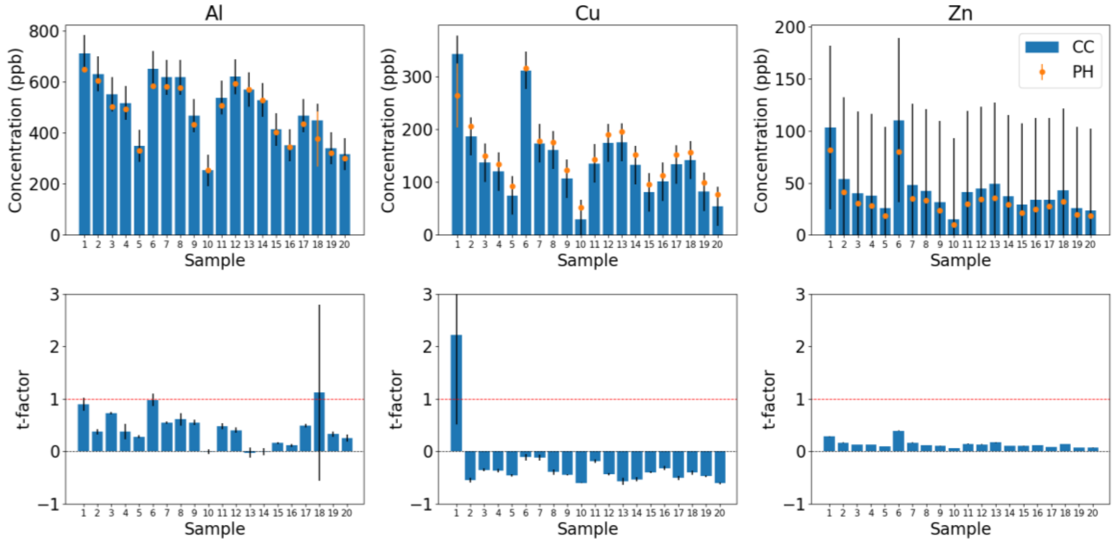

Supplementary Figure S2. Comparison between compressed calibration (CC) and direct ICP-MS peak hopping (PH) results for the concentrations of elements Al, Cu and Zn in the diluted samples directly measured in TAIL-UC, for the MBM medium. In the first row, CC results are shown as blue bars and the corresponding estimated standard deviation in black error bars. The PH results are shown as orange circles, with corresponding estimated standard deviations, although obscured by the circles in most cases. On the second row, blue bars indicate the *t*-factor computed as the difference between the CC and PH estimated concentrations, divided by the corresponding estimated standard deviations for CC. The error bars indicate, in this case, the estimated standard deviations for PH divided by the estimated standard deviations for CC.

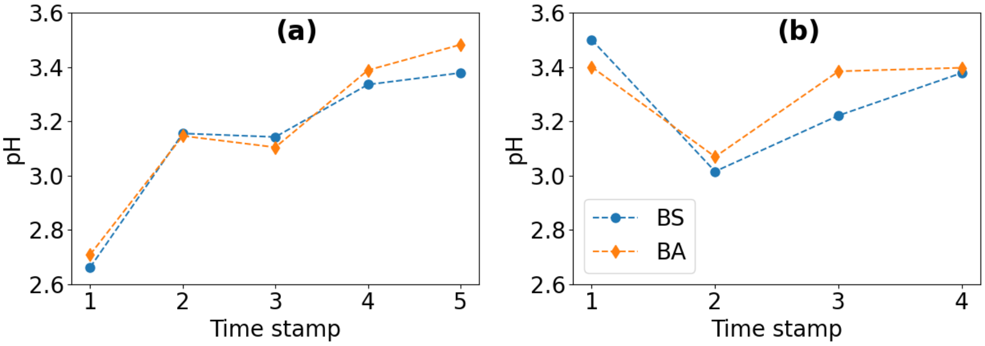

Supplementary Figure S3. Results of pH measurements during the column bioleaching assay with R2Ab medium for biostimulation and bioaugmentation (with strain B2A2W2 growth) conditions, in effluent water **(a)** and pore water samples **(b)**.
